# Rectified Cell Migration on Saw-Like Micro-Elastically Patterned Hydrogels with Asymmetric Gradient Ratchet Teeth

**DOI:** 10.1371/journal.pone.0078067

**Published:** 2013-10-17

**Authors:** Satoru Kidoaki, Hiroyuki Sakashita

**Affiliations:** 1 Research Field of Biomedical and Biophysical Chemistry, Institute for Materials Chemistry and Engineering, Kyushu University, Fukuoka, Japan; 2 Graduate School of Engineering, Kyushu University, Fukuoka, Japan; Brandeis University, United States of America

## Abstract

To control cell motility is one of the essential technologies for biomedical engineering. To establish a methodology of the surface design of elastic substrate to control the long-range cell movements, here we report a sophisticated cell culture hydrogel with a micro-elastically patterned surface that allows long-range durotaxis. This hydrogel has a saw-like pattern with asymmetric gradient ratchet teeth, and rectifies random cell movements. Durotaxis only occurs at boundaries in which the gradient strength of elasticity is above a threshold level. Consequently, in gels with unit teeth patterns, durotaxis should only occur at the sides of the teeth in which the gradient strength of elasticity is above this threshold level. Therefore, such gels are expected to support the long-range biased movement of cells via a mechanism similar to the Feynman-Smoluchowski ratchet, i.e., rectified cell migration. The present study verifies this working hypothesis by using photolithographic microelasticity patterning of photocurable gelatin gels. Gels in which each teeth unit was 100–120 µm wide with a ratio of ascending:descending elasticity gradient of 1:2 and a peak elasticity of ca. 100 kPa supported the efficient rectified migration of 3T3 fibroblast cells. In addition, long-range cell migration was most efficient when soft lanes were introduced perpendicular to the saw-like patterns. This study demonstrates that asymmetric elasticity gradient patterning of cell culture gels is a versatile means of manipulating cell motility.

## Introduction

Cell motility is fundamental to the dynamic behavior of living tissues, and plays an essential role in the physiological and pathological processes such as morphogenesis [1-4], inflammation [5,6], wound healing [7,8], and tumor metastasis [9,10]. For tissue engineering and regeneration [11], the recruitment and localization of cells in regenerating tissues needs to be controlled by manipulating cell migration in principle [12]. Controlled cell motility is essential *in vivo*; thus also required for biomedical engineering.

*In vivo* system, cell motility is controlled by external stimuli that induce directional cell movement. These so-called taxis behaviors include chemotaxis [13], phototaxis [14], galvanotaxis [15], geotaxis [16], haptotaxis [17], and durotaxis/mechanotaxis [18-20]. These intrinsic responses of living cells can be controlled by gradient factors such as soluble chemicals, light, electrochemical potential, gravity, surface-fixed chemicals, and culture matrix rigidity, respectively. To establish the technology to control cell motility at will, it is essential to manipulate cell taxis behaviors through the programmed setting of such the extracellular operation parameters.

Of the aforementioned cellular taxis behaviors, haptotaxis and durotaxis can be artificially controlled by modulating the characteristics (e.g., surface chemistry and bulk mechanics) of the extracellular scaffold, matrix or substrate, thus useful for the sake of functional design of the biomaterial surfaces to manipulate cell migrations. Haptotaxis drives long-range cell migration along gradients of surface-fixed haptoattractants, such that cells migrate towards regions with higher concentrations of these factors in the millimeter scale [21-23]. On the other hand, durotaxis (mechanotaxis towards more rigid regions) is induced near the elasticity boundary having sharp elasticity jump over a certain threshold of elasticity gradient strength in single cell adhered area (smaller than ca. 50-100 μm) [24,25]. Although durotaxis can be controlled by modulating the gradient strength of elasticity boundary, the resulting biased movement of cells is restricted around this boundary, and cells tend to move randomly in other regions. Therefore, if elastic substrates are to be used to control long-range cell movement, the substrate must have multiple elasticity boundaries. In this study, to establish a methodology of the surface design of elastic substrate to control the long-range cell movements, we developed a cell culture hydrogel to support the long-range durotaxis which is beyond distance limitation on the single cell adhered area and enable to reach to the millimeter scale of distance.

**Figure 1:**
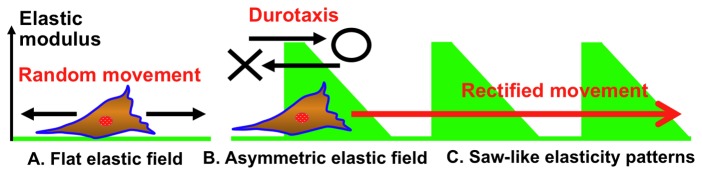
Schematic diagram showing how saw-like asymmetric elasticity patterns are hypothesized to induce rectified cell migration. (a) Cells move randomly when substrate elasticity is homogeneously distributed. (b) On a surface with asymmetric elasticity, cells move by durotaxis towards regions with high elasticity across boundaries that have an elasticity gradient above a threshold level. Biased cell migration does not occur at boundaries that have an elasticity gradient below this threshold level. (c) Cells that migrate across the region with peak elasticity do not migrate back to the less rigid area. Instead, they enter the adjoining unit area. This process can lead to long-range cell migration via a mechanism similar to that of the Feynman ratchet.

Our strategy to control the long-range durotaxis was to design gels with asymmetric elasticity patterns that would rectify random cell movement (Figure 1). In this model, gels with a tooth-like pattern are generated in which elasticity sharply increases and then gradually declines. Cells should show biased movement toward the region that has a sharp increase in elasticity (i.e., the elasticity gradient strength is above a threshold level) and move away from the region in which elasticity gradually declines (i.e., the elasticity gradient strength is below a threshold level). This should induce biased long-range cell movement via a mechanism similar to the Feynman-Smoluchowski ratchet [26]. For this trial, we used photocurable styrenated gelatins to fabricate micro-elastically patterned gels with asymmetric tooth-like patterns. Long-range cell migration on gels with various peak elasticities and unit widths was determined. Gels in which each teeth unit was 100–120 µm wide with a ratio of ascending:descending elasticity gradient of 1:2 and a peak elasticity of ca. 100 kPa supported the efficient rectified migration of 3T3 fibroblast cells. In addition, long-range migration was most efficient when soft lanes were introduced perpendicular to the saw-like patterns. The mechanism by which these gels support long-range cell migration and the applications of these gels is discussed.

## Materials and Methods

### Preparation of photocurable sol solution

Elasticity-tunable hydrogels were prepared from photocurable styrenated gelatin (StG). StG was synthesized as described previously [27]. The sol solution of StG was prepared as follows: (1) StG (30 wt%) and sulfonyl camphorquinone (Toronto Research Chemicals, ON, Canada; 1.5 wt% of gelatin) were dissolved in phosphate buffered saline (PBS). (2) The solution was centrifuged (MX-301; TOMY, Tokyo, Japan) at 17,800 × g for 1 hr to pellet coagulated colloidal particles. (3) The clarified sol solution was mildly aspirated to remove dissolved gas, conditioned for 10 min using a deforming agitator (MX-201; THINKY, Tokyo, Japan), and stored at -20°C. The sol solution was equilibrated at 45°C in a nitrogen atmosphere for 30 min prior to use for photolithography.

### Photolithographic micro-elasticity patterning of gelatinous gels

**Figure 2:**
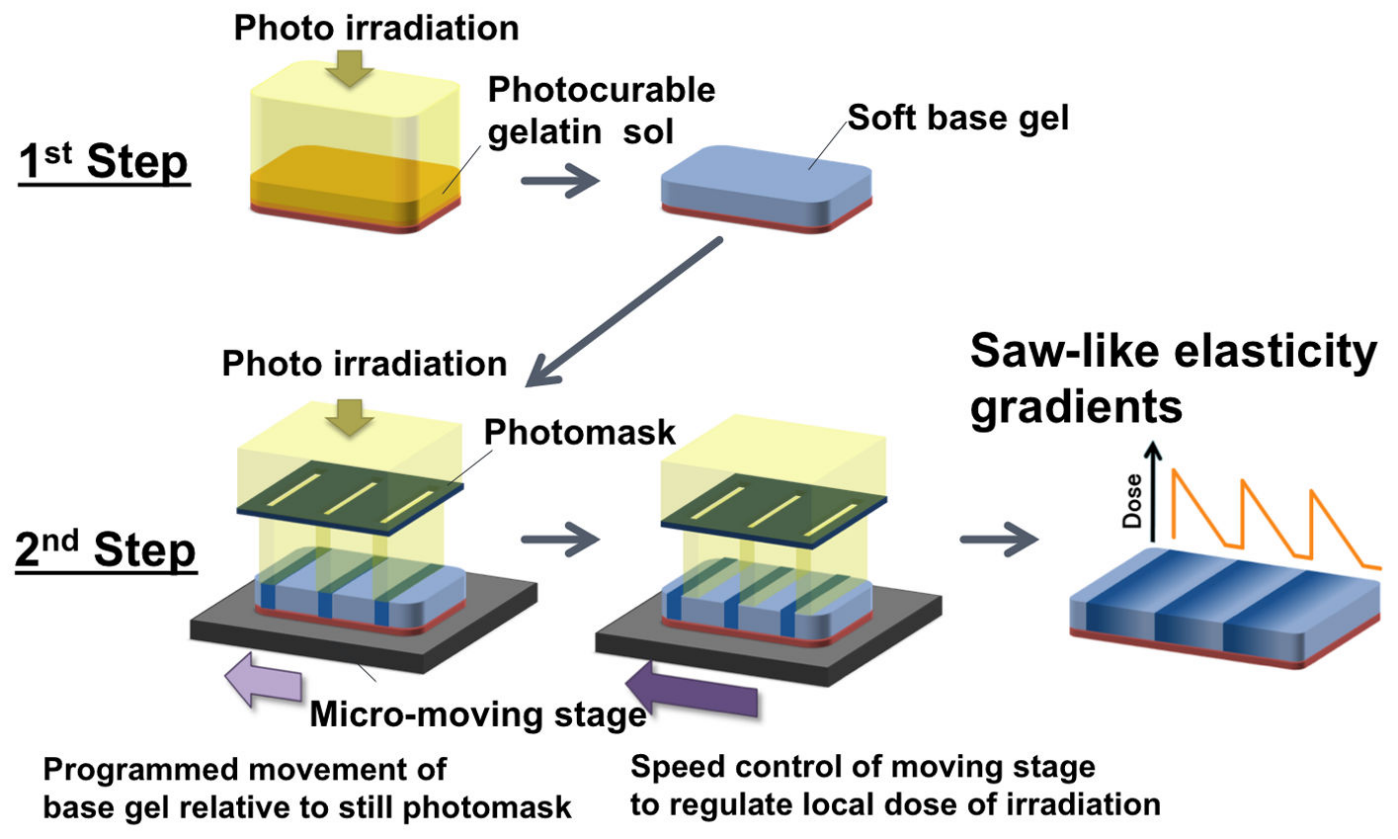
Schematic diagram showing how cell adhesive hydrogels with saw-like asymmetric elasticity patterns are fabricated. In the first step, a soft base gel is prepared by irradiation of a photocurable sol of styrenated gelatin with visible light. In the second step, the base gel is photo-irradiated through slits in a photomask for a defined period of time. The gel is continuously displaced in a perpendicular direction to the photomask at a defined speed using a computer-assisted X-Y movement stage. The duration of irradiation is set so that the StG sol surface is irradiated asymmetrically.

Photolithographic micro-elasticity patterning of the StG sol solution was performed as follows: The sol solution was spread between a glass substrate modified with vinyltrimethoxysilane (Tokyo Chemical Industry Co. Ltd., Tokyo, Japan) and a normal glass substrate coated with poly(*N*-isopropylacrylamide) (PNIPAAm, Sigma Aldrich Co. MO, USA), and the sample was placed on a hot plate at 45°C. PNIPAAm was used to ensure that photocured StG gel could be easily removed from the normal glass substrate by treating it with an aqueous solution below the lower critical solution temperature. A soft base gel was first prepared by irradiating the entire sample with visible light (100 mW/cm^2^ at 488 nm) from behind the vinyl-silanized glass (Figure 2) for different times. Next, local photoirradiation (120 mW/cm^2^ at 488 nm) of the soft base gel was performed through a striped-patterned photomask with slits and light blockers of various widths (slit/shade: 60/140, 40/200, 60/180, and 30/150 µm) and programmed movement (in the X-direction) on a computer-assisted micro-moving stage (Kohzu Precision Co., Ltd, Kanagawa, Japan) using a custom-designed reduced projection-type photolithographic system as previously reported [24,25]. The speed and distance that the base gels moved during the programmed irradiation are described in Table 1. In this system, a 2 × objective (NA 0.1, Nikon Corporation, Tokyo, Japan) and a metal halide light source (MME-250; Moritex, Tokyo, Japan) were used. Light intensity was measured using a laser power meter (HP-3; Pneum Co., Ltd. Saitama, Japan). Finally, the gels were detached from the PNIAAm-coated normal glass substrate and thoroughly washed with PBS at room temperature to completely remove the adsorbed PNIPAAm.

**Table 1:** Operation parameters for photolithographic micro-elasticity pattering.

Gel	PhotomaskSlit/Shade (µm)	1st step irradiation (s)	Stage moving speed 1 (µm/s)	Stage moving distance 1 (µm)	Stage moving speed 2 (µm/s)	Stage moving distance 2 (µm)
A1	60/140	65	0.66	30	1.33	30
A2	60/140	65	0.57	30	1.14	30
A3	60/140	70	0.44	30	0.88	30
B1	40/200	90	0.44	40	0.88	40
B2	40/200	100	0.14	40	0.30	40
B3	60/180	105	0.11	30	0.27	30
PSL(+)	X:30/150; Y:50/200	65	0.20	15	0.40	15

* 2^nd^ step irradiation was performed during stage moving process.

### Measurement of the surface elasticity distribution of micropatterned StG gels

The surface elasticity of the photocured StG gel was determined by nanoindentation analysis using atomic force microscopy (AFM). Force-indentation (*f-i*) curves of the gel surface in PBS were measured by AFM (NVB100; Olympus Optical Co. Ltd., Tokyo, Japan; AFM controller & software, Nanoscope IIIa; Veeco Instruments, CA, USA) with a commercial silicon-nitride cantilever with a nominal spring constant of 0.02 N/m and an integrated pyramidal tip (OMCL-TR400PSAHW, Olympus Optical Co. Ltd., Tokyo, Japan). A frequency of 1 Hz was chosen for the tip approach/retract cycle to minimize noise fluctuation within a single *f*–*i* curve. The surface Young’s moduli were evaluated from the *f-i* curves by nonlinear least-squares fitting to the Hertz model in the case of a conical indenter [eq. (1)] (semi-vertical angle (α): 30°, Poisson ratio (µ): 0.5) [28-30].

$$F=\frac{2tan\alpha E}{\pi (1-\mu^{2})}\delta^{2}$$ (1)

The distribution of Young’s moduli on the micro-elastically patterned gels was obtained by measuring the manual force-volume at a resolution of 10 µm.

### Cell culture

The mouse fibroblast cell line (NIH/3T3) was purchased from Dainippon Sumitomo Pharmaceutical Co. Ltd. (Osaka, Japan) and cultured in Dulbecco’s modified Eagle’s medium (Gibco BRL, Grand Island, NY, USA) supplemented with 10% fetal bovine serum (Gibco BRL), 3.5 g/l glucose, 2 mM L-glutamine, 50 units/ml penicillin, and 50 µg/ml streptomycin. Cells were maintained on tissue culture polystyrene dishes at 37°C in an atmosphere of 5% CO_2_ in a humidified incubator.

### Time-lapse observation of cell migration

Cell migration on micro-elasticity patterned gel surfaces was monitored using an automatic time-lapse microscope (Bio-Revo; Keyence Corporation, Osaka, Japan). Cells were imaged at 37°C in a humidified chamber containing 5% CO_2_ (TOKAI HIT company, Shizuoka, Japan). Prior to imaging, cells were seeded on the gel at a density of 1.5 × 10^3^ cells/cm^2^ and cultured in Dulbecco’s modified Eagle’s medium supplemented with 10% fetal bovine serum in an atmosphere of 5% CO_2_ for 8 hr. Images were captured every 15 min for 24–30 hr. The coordinates of 20–30 cells and their migratory tracks were measured using ImageJ software.

### Confocal microscopy

The surface topography of the patterned gels was observed using a confocal laser scanning microscope (LSM510META, Carl Zeiss, Oberkochen, Germany). The gel samples were adsorbed and stained with fluorescein-conjugated albumin, and confocal cross-sectional images were acquired.

## Results

### Fabrication of saw-like micro-elastically patterned gels with asymmetric gradient teeth

**Figure 3:**
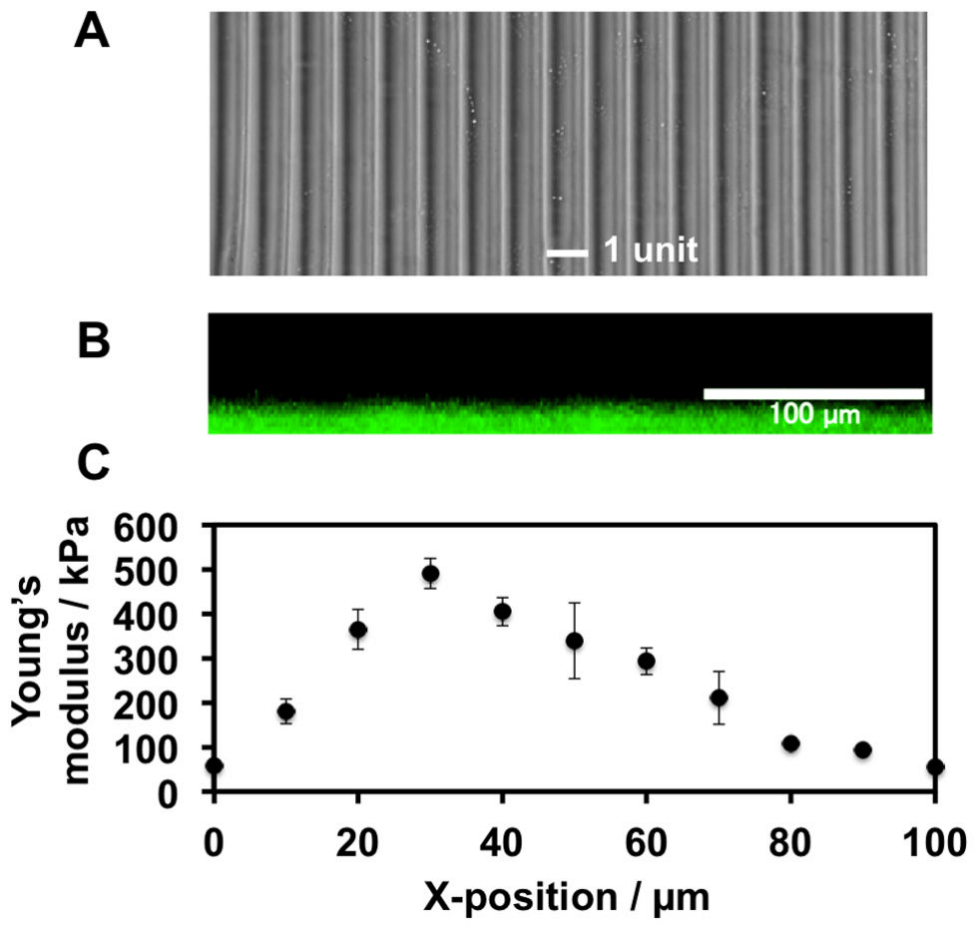
A representative gel with a saw-like asymmetric elasticity pattern. Width of each unit: 100 µm. (a) Phase contrast microscopy image of the gel surface. (b) Cross-sectional observation of the gel surface by confocal laser scanning microscopy. (c) Distribution of Young’s modulus in a single unit. Preparation conditions: first irradiation step, 70 s; second irradiation step, stage moved at a velocity of 0.44 µm/s between 0 and 19 µm and then at 0.88 µm/s between 20 and 59 µm using a photomask with a 60 µm-wide slit and a 140 µm-wide shade.

We fabricated saw-like elastically patterned gels with asymmetric gradient teeth by a two-step photoirradiation lithographic method using a micro-moving stage (Fig. 2). In this method, local doses of photoirradiation are delivered to specific regions of the gel surface through slits in a photomask. The regions that are irradiated are determined by the movement of the pre-prepared soft base gel on the stage relative to the static photomask. The micro-moving stage displaces the gel in the X-direction perpendicular to the striped patterns of light projected by the photomask. The programmed distances and speeds moved by the stage govern the amount of displacement. In this way, different regions of the gel surface are exposed to different amounts of photoirradiation. Figure 3 shows a representative gel prepared using this technique, which has a saw-like pattern with 100 µm-wide unit teeth and in which the width ratio of ascending:descending elasticity gradients of each unit was 1:2. To produce this gel, the stage was moved in the X-direction at a velocity of 0.44 µm/s between 0 and 30 µm and then at 0.88 µm/s between 30 and 60 µm, using a photomask with a 60 µm-wide slit and a 140 µm-wide light blocker in half reduction mode (i.e., the final projected pattern comprised 30 µm/70 µm slit/shade). This technique generated a gel with a saw-like elastically patterned surface (Figure 3A) and a smooth topography, as confirmed by cross-sectional observation of the gel by confocal microscopy (Figure 3B). When gels were continuously irradiated through 30 µm-wide moving slits using the aforementioned program settings, there was a sharp increase in Young’s moduli in the first third of the tooth unit pattern, and a gradual decrease in the latter two thirds (Figure 3B). Under these conditions, the maximum elasticity was ca. 500 kPa and the elasticity of the soft base was ca. 50 kPa (the A3 condition).

**Figure 4:**
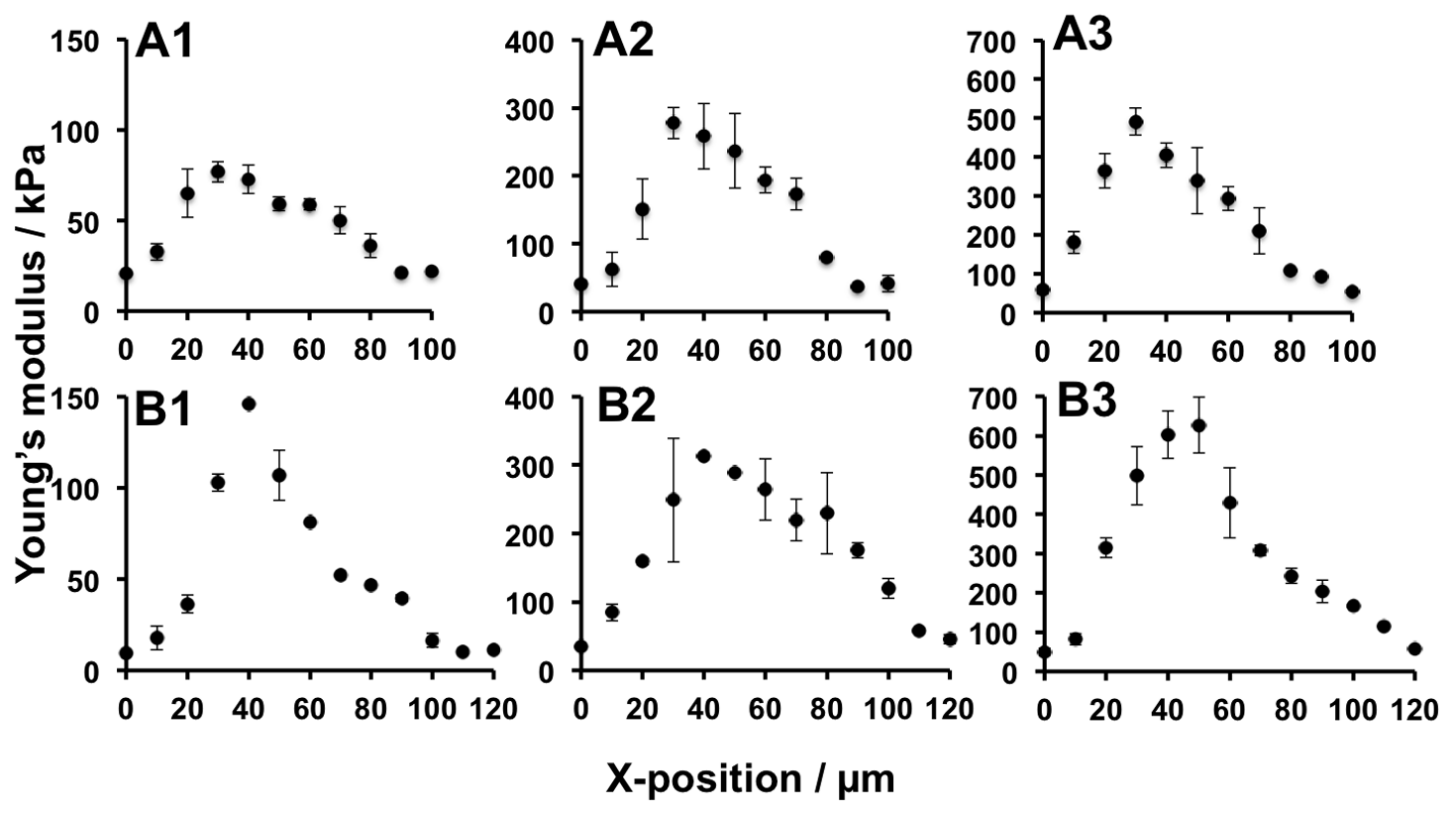
Distribution of Young’s moduli in gels with different unit sizes and peak elasticities. The width of each unit in series A and B was 100 µm and 120 µm, respectively. Series 1, 2, and 3 had a peak elasticity of ca. 100 kPa, 300 kPa, and 500 kPa, respectively. The ratio of ascending:descending elasticity gradients in each unit was 1:2, i.e., the X-position of the peak of elasticity was located at ca. 30 µm and 40 µm in series A and B, respectively. The conditions used to prepare each of the gels are described in Table 1.

To investigate the elasticity distribution in the asymmetric gradient teeth that can induce long-range cell migration, gels with six types of unit teeth were prepared (Fig. 4). The units were designed to be 100 µm (series A) or 120 µm (series B) wide, and the maximum elasticity was 1) ca. 100 kPa, 2) ca. 300 kPa, or 3) ca. 500 kPa. The elasticity of the soft base gel was 50 kPa. We used these gels because we have previously shown that gelatinous gels with similar features induce durotaxis [24]. The ratio of the ascending:descending elasticity gradients around the elasticity peak was fixed at 1:2. This ratio was used to ensure that the increase in elasticity per single adhered cell area in the ascending slope was sufficiently sharp, as established in our previous study [25]. This is essential to induce fibroblast durotaxis. These gels were produced by programming the velocity and displacement of the moving stage to deliver asymmetric doses of photoirradiation. The conditions used in each experiment are described in the figure legends. The surface topography of the A1, A2, and B1–3 gels was smooth, similar to that of the A3 gel (Figure 3B) (data not shown).

### Evaluation of cell migration on gels with asymmetric elasticity

**Figure 5:**
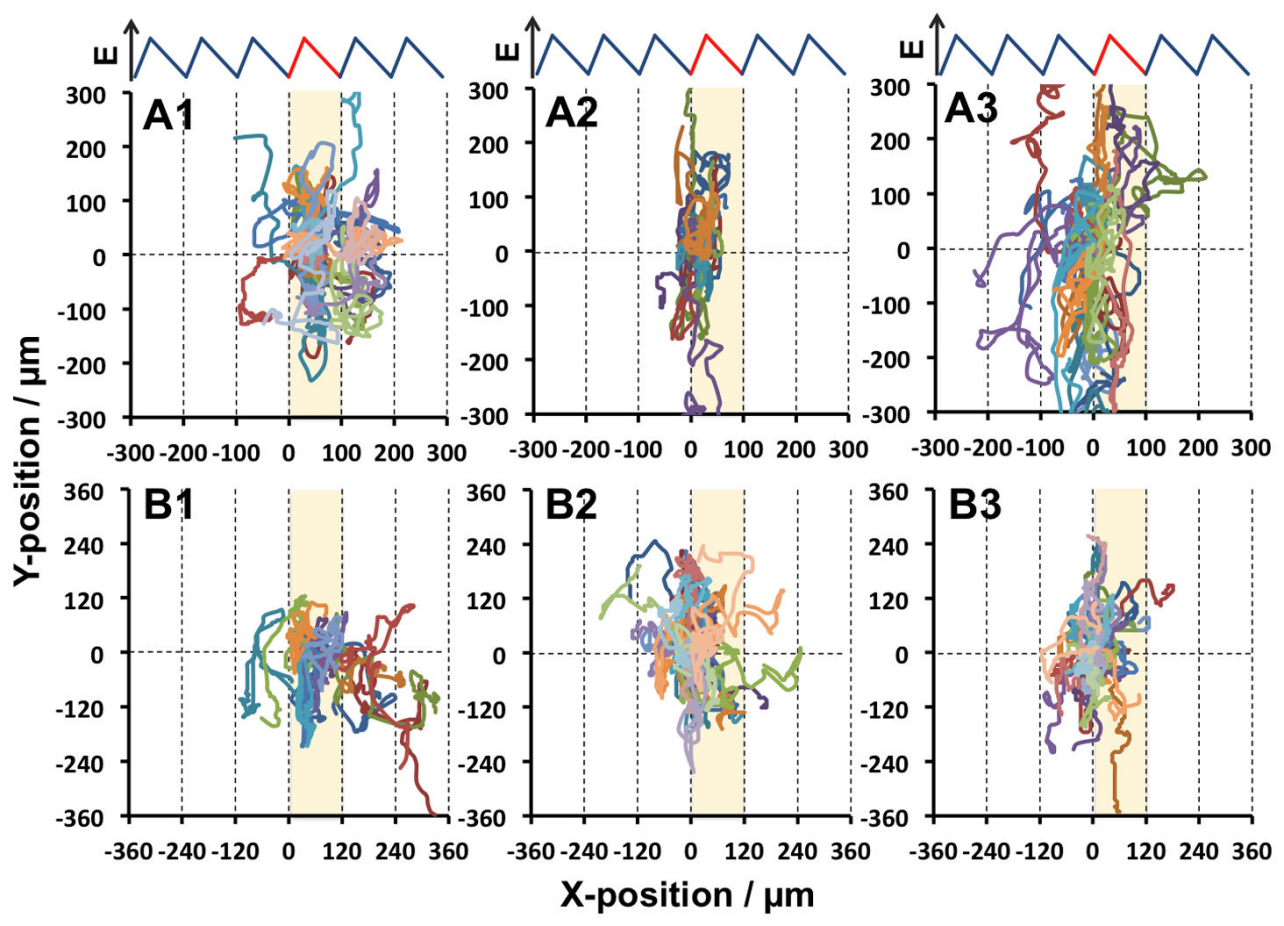
Trajectories of 3T3 fibroblasts cultured on A1-A3 and B1-B3 gels. Time-lapse microscopy was performed for 30 hr and images were acquired at 15 min intervals. The starting position of each cell trajectory was mapped to the origin of the graph. A band of single unit including the origin is highlighted. n=40 cells.

To determine whether durotaxis occurs on any of the saw-like micro-elastically patterned gels, we examined the migration of fibroblasts on each of the gels. Cells were observed by time-lapse microscopy and images were acquired at 15 min intervals for 30 hr. Cells were seeded on the gels at low density to avoid cell-cell contact, which disturbs the mechanics of cell-substrate interactions and may therefore interfere with migration. Figure 5 shows the trajectories of the fibroblasts on the gels. In each graph, the results of three time-lapse observations are superimposed. The starting position of each cell trajectory was fixed to the origin of the graphs, and the unit of the saw-like pattern at which the cell trajectory started is colored. Of the six gel types, biased cell trajectories toward the right, which is the anticipated direction of durotaxis in this system (Figure 1), was observed over two and three teeth on A1 and B1 gels, respectively, during the 30 hr observation period.

**Figure 6:**
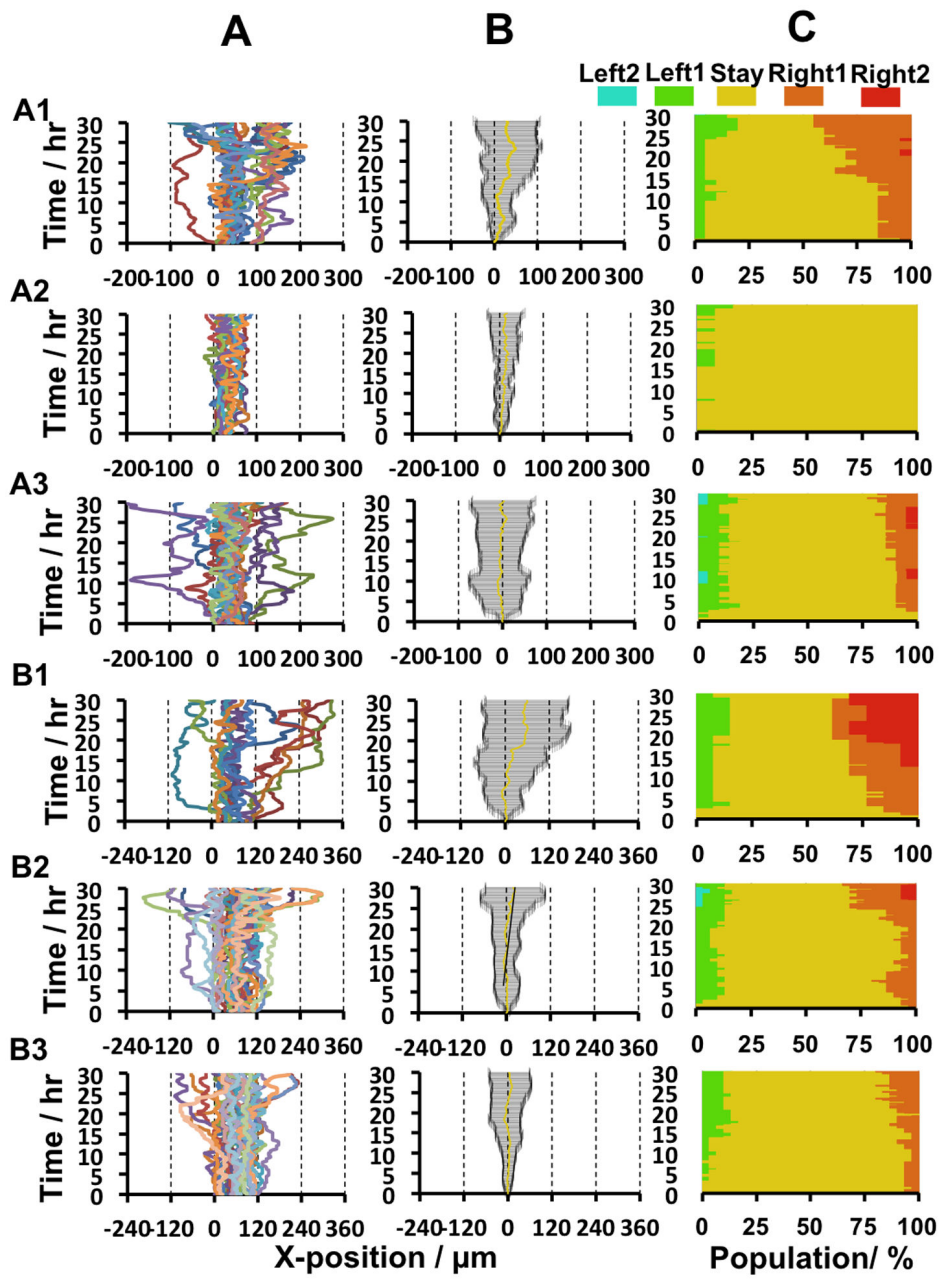
Quantitative analyses of observed cell trajectroies. a) Time-course analysis for X trajectory of each cell observed, b) ensemble averaged displacement in the X-direction with standard deviation, c) a heat map of the regions of the gel to which cells migrated.

To quantitatively characterize rectified cell migration, the following time-courses were plotted: the observed X-trajectory of each cell (Figure 6A), ensemble averaged displacement in the X-direction (Figure 6B), and heat maps of the regions on each gel to which the cells migrated (Figure 6C). Biased time-course trajectories toward the right were confirmed for A1 and B1 gels, which had 100 µm- and 120 µm-wide unit patterns, respectively, and a peak elasticity of ca. 100 kPa. Gel A1 clearly induced biased cell migration over one tooth unit, and the percentage of cells in the first-adjoining unit increased during the 30 hr observation period (Figure 6C, “Right 1” population). Gel B1 also induced biased cell migration, as indicated by an increase in the percentage of cells in the second-adjoining unit (Figure 6C, “Right 2” population). Such cell migration in the X-direction was not detected on any of the other gels, even though these gels had asymmetric elastic gradients. Instead, cells exhibited biased migration in the Y-direction on these gels. These data show that the basic conditions required to induce long-range rectified migration of fibroblasts are as follows: 100–120 µm-wide units, a ratio of ascending:descending elasticity gradients of 1:2, and a peak elasticity of ca. 100 kPa.

### Long-range rectification of cell migration on a gel with soft lanes perpendicular to the teeth

**Figure 7:**
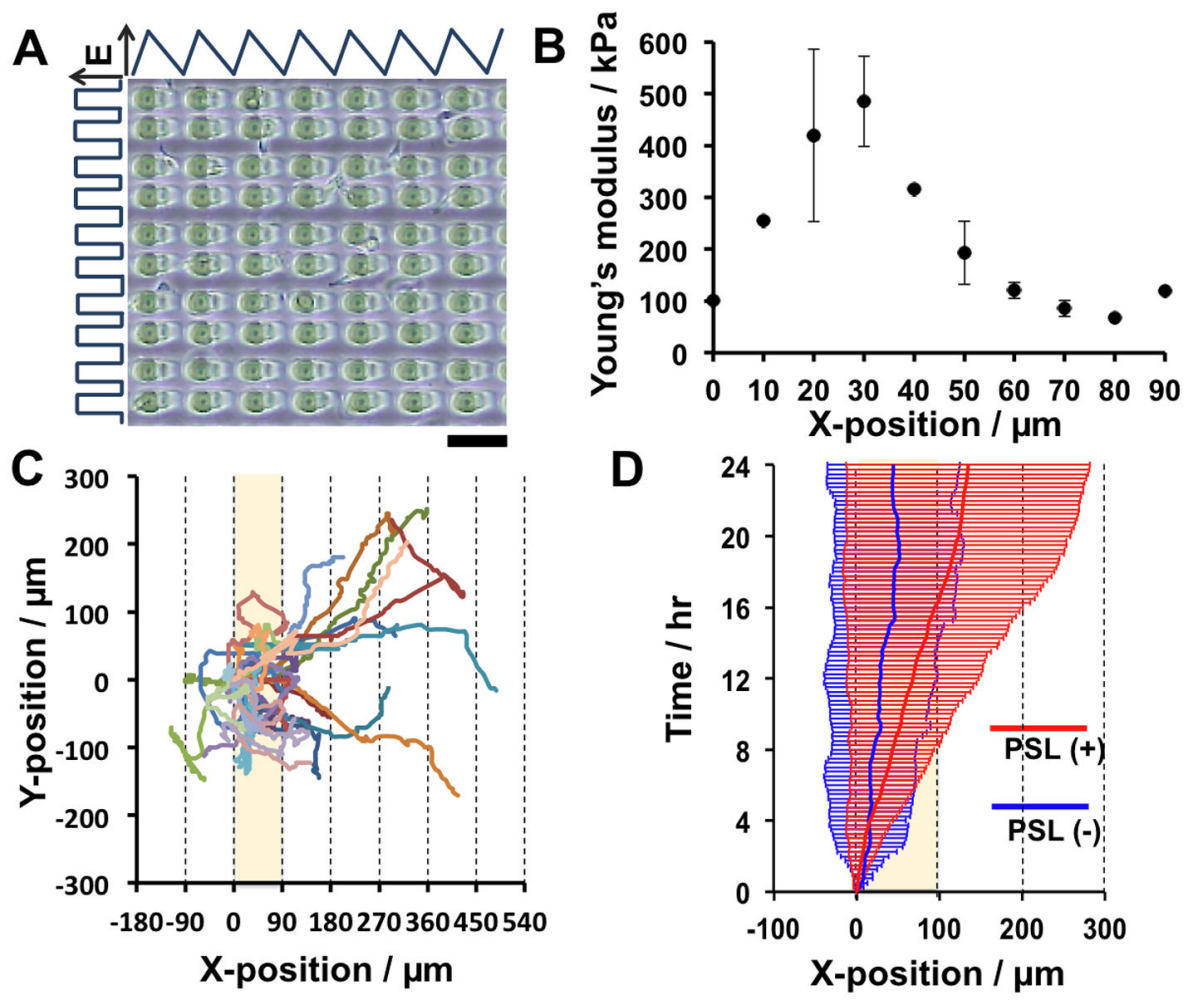
Long-range cell migration on gels with saw-like asymmetric elasticity patterns and perpendicular soft lanes (PSLs). The conditions used to prepare the gels are described in Table 1. (a) Phase contrast microscopy image of the gel. Sale bar: 100 µm. (b) Distribution of Young’s modulus in a single unit of the gel. Unit size: 90 µm. Elasticity peak: ca. 500 kPa. (c) Trajectories of 3T3 fibroblasts cultured on gels with PSLs. Cells were observed by time-lapse microscopy for 24 hr and images were acquired at 15 min intervals. (d) Time-course of ensemble averaged trajectories with standard deviation in the presence (red) and absence (blue) of PSLs. n=30 cells.

Unwanted biased cell migration in the Y-direction, due to small topographic patterns of swelling of the soft base gel, was observed on all of these gels (see Figure 3B). Suppression of such migration would be expected to enhance long-range cell migration in the X-direction. To accomplish this, perpendicular soft lanes (PSLs) were introduced into the saw-like patterns. Figures 7A and B show a phase contrast microscopy image of this gel and its elasticity distribution, respectively. The gel was designed to have a peak elasticity of ca. 500 kPa, 90 µm-wide unit teeth, and 20 µm-wide PSLs. The trajectories of cells grown on gels with PSLs (+) exhibited marked long-range migration toward the right over 4 unit teeth and a displacement of up to 450 µm during the 24 hr observation period (Figure 7C) compared with cells grown on gels without PSLs (-) (Figure 7D). These data show that saw-like micro-elastically patterned gels with PSLs most effectively induce long-range cell migration.

## Discussion

In the present study, we proposed that saw-like micro-elastically patterned hydrogels with asymmetric gradient ratchet teeth may rectify random cell movement and induce long-range migration *in vitro*. This hypothesis was based on the following characteristics of durotaxis: 1) durotaxis is only induced at regions with elasticity gradients above a threshold level [25]; 2) cells migrate randomly in regions with elasticity gradients below this threshold level; and 3) cells do not return to less rigid regions once they have migrated across regions with elasticity gradients above the threshold level. This means that gels with an asymmetric elasticity gradient, in which one side of each unit has a high elasticity gradient and the opposite side has a low elasticity gradient, could induce long-range cell migration and prevent random cell movements.

In the first summary, gels in which each unit was 100–120 µm wide with a ratio of ascending:descending elasticity gradients of 1:2 and a peak elasticity of ca. 100 kPa were found to support the rectified cell migration (A1 or B1 gels). Moreover, rectification of cell migration occurred more efficiently on gels with 120 µm-wide teeth (B1 gels) than on gels with 100 µm-wide teeth (A1 gels). This is because cell migration in the Y-direction occurred less frequently on B1 gels than on A1 gels. The other gel types did not support rectified cell migration, with cells tending to migrate in the Y-direction. 

The reason why cells migrated better on certain gels is due to the balance between two parameters: the width of the diffuse side of the elasticity boundary (WDB) and the elasticity peak height (EPH). When WDB is smaller, or when EPH is larger, this tends to force cells to move towards the area of greatest elasticity, which enhances migration in the Y-direction and prevents rectified migration in the X-direction. This may explain why cell migration in the Y-direction occurred more frequently on A1 gels than on B1 gels, and why cell migration in the X-direction was poor on A2, A3, B2, and B3 gels (Figures 4 and 5). The highly efficient rectification of cell migration on B1 gels can be attributed to this gel having a larger WDB (> ca. 70 µm) and lower EPH (< ca. 100 kPa) than the other gels.

In principle, however, a smaller WDB and a higher EPH is expected to be required for more efficient long-range cell migration if the biased migration in Y-direction is appropriately prevented, because the former allows cells to find the elasticity peak in the neighboring tooth unit and the latter is favorable for the induction of durotaxis. Therefore, a gel with a WDB of 60 µm, an EPH of 500 kPa, and with PSLs was designed (Figure 7). As expected, cell migration was most efficient on this gel.

In the second summary, let us stress that the method of controlling cell migration by micro-elasticity patterning developed in this study is significant and has many potential applications. Concerning the methods to induce directed cell migration, those based on the surface chemistry and topography of different cell substrates have been developed previously. Jiang et al. reported electrochemical patterning and dynamic on/off manipulation of asymmetrically-patterned adhesive regions of single cells, and investigated the role of cell polarization induced by this patterning in determining the direction of successive cell movements [31]. Chemical patterning of asymmetric tear-like cell adhesive regions [32] and geometrically-patterned wells of connected triangles [33] demonstrated that ratchet-like patterns induce rectified cell migration. Cell culture surfaces with topographic asymmetric or anisotropic patterns also effectively prevent random cell movement and induce long-range rectified cell migration [34,35]. For these reports, the present study is the first to use micro-elasticity patterning of asymmetric gradient ratchet teeth to induce long-range rectified cell migration. One of the most important features of the hydrogels described herein is that the elasticity distribution is well-defined for gels with specific surface chemistries and topographies. These features were described in detail for patterned StG gels in our previous study [25]. 

Besides in this system, cells receive different mechanical signals from a specific region of the gel in their spontaneous moving process. These signals affect not only cell motility but also other cell functions, including proliferation and differentiation [36-40]. In theory, the mechanical signals that migrating cells receive from the substrate could be programmed in this system through designing the elasticity patterns, which would enable coupled control of cell migration and other cellular functions. This system could also be used to investigate the mechanics of cell motility by enabling systematic traction force microscopy analysis [41,42] of surfaces with well-defined elasticity gradients. The generation of materials with well-defined micro-elasticities, such as the gels described in this study, is expected to be useful not only for cell manipulation technologies including the above-mentioned mechanosignal-regulating biomaterials and the separations of different type of cells (see our preliminal results shown in Figure S1), but also for basic cell research especially on cell mechanobiology. We are currently investigating the application of such materials in both these areas.

## Conclusion

In the present study, to establish a methodology of the surface design of elastic substrate to control the long-range cell movements which serves to recruit and reposition the cells in regenerating/engineering tissues, we proposed a hypothesis and strategy to control long-range durotaxis, i.e., the application of saw-like micro-elastically patterned hydrogels with asymmetric gradient ratchet teeth to prevent random cell movement. Durotaxis only occurs at the side of the teeth at which elasticity increases sharply, and therefore biased cell movement occurs in a manner similar to the Feynman- Smoluchowski ratchet. Gels in which each unit is 100–120 µm wide with a ratio of ascending:descending elasticity gradients of 1:2 and a peak elasticity of ca. 100 kPa support long-range durotaxis. In addition, the efficiency of long-range durotaxis is increased when perpendicular soft lanes are introduced into the saw-like patterns to suppress the migrations along Y-axis. The moving ditance reaches to ca. 500 µm for 24hrs culture, suggesting cell migration in millimeter scale is enough possible for long-term culture. Asymmetric elasticity gradient patterning was first shown to be a versatile means of manipulating cell motility.

## Supporting Information

Figure S1 **Migration responses of different type of cell on the saw-like micro-elastically patterned gels.** Smooth muscle cells (rabit, primary) and human mesenchymal stem cells (purchased from Ronza) were cultured for 24hr on the gels of A1 condition, and compared with the behaviors of 3T3 fibroblasts. Observed cell trajectories are shown.
(PDF)
