# Supplementary material for: Rectified Cell Migration on Saw-Like Micro-Elastically Patterned Hydrogels with Asymmetric Gradient Ratchet Teeth
Source: PLoS One. 2013 Oct 17;8(10):e78067. doi: 10.1371/journal.pone.0078067 (PMC3798417; doi:10.1371/journal.pone.0078067)

Figure S1

Both rSMC and hMSC did not show the rectified migrations, while 3T3 showed biased migrations reaching 2 units of patterns during 24hr culture. To induce the rectified long range movements for SMC and MSC, optimization of pattern size and elasticity distribution should be required, because different type of cell has its intrinsic cell morphology and motility. On the other hand, based on such difference in motility responses, cell separation and screening would be possible. In the present case, each three type of cell showed marked different motility: rectification for 3T3, trapped behaviors in single unit of pattern for rSMC, and long range migration along Y axis for hMSC.

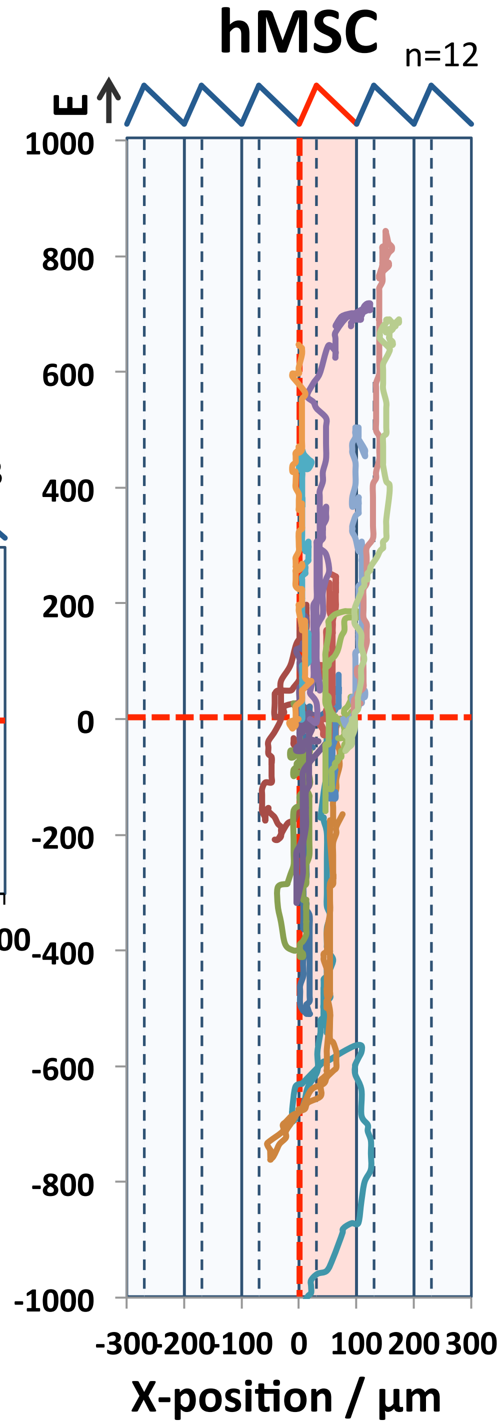

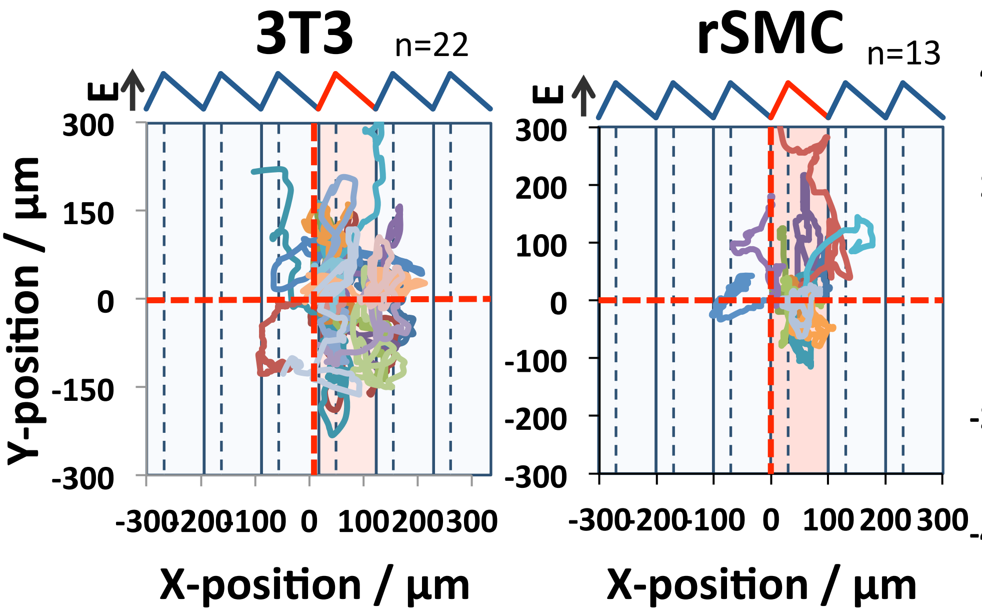

Supplement: Figure S1 — Migration responses of different type of cell on the saw-like micro-elastically patterned gels. Smooth muscle cells (rabit, primary) and human mesenchymal stem cells (purchased from Ronza) were cultured for 24hr on the gels of A1 condition, and compared with the behaviors of 3T3 fibroblasts. Observed cell trajectories are shown. (PDF) [file pone.0078067.s001.pdf]
